# Primary Immune Thrombocytopenic Purpura (ITP) and ITP Associated with Systemic Lupus Erythematosus: A Review of Clinical Characteristics and Treatment Modalities

**DOI:** 10.1155/2024/6650921

**Published:** 2024-03-01

**Authors:** Krishna Prasad Bashyal, Sangam Shah, Calvin Ghimire, Shravya Balmuri, Pradip Chaudhary, Sandip Karki, Anuj Krishna Poudel, Ashbina Pokharel, Vishal Devarkonda, Samina Hayat

**Affiliations:** ^1^McLaren Health Care Corp, 401 South Ballenger Hwy, Flint 48532, USA; ^2^Tribhuvan University, Institute of Medicine, Maharajgunj, Kathmandu 44600, Nepal; ^3^Louisiana State University Health Sciences Centre Shreveport, Louisiana, Shreveport, USA 71103-4228; ^4^Hurley Medical Center, Flint, MI, USA; ^5^Greater Baltimore Medical Center, Baltimore, USA; ^6^William Beaumont Hospital, Royal Oak, Michigan 48073, USA

## Abstract

Immune thrombocytopenic purpura (ITP) is an immune-mediated disorder characterized by the destruction of platelets and megakaryocytes due to autoantibodies against the platelet surface proteins. ITP without any apparent cause of thrombocytopenia is defined as primary ITP, and ITP in the setting of SLE is secondary ITP, which can be diagnosed after excluding other causes of thrombocytopenia by history, physical examination, and laboratory testing. Patients with ITP associated with SLE have higher median platelet count and less bleeding manifestations compared to the patients with primary ITP. It can be very challenging to diagnose primary ITP in SLE patients as other causes of thrombocytopenia including drug-induced thrombocytopenia, antiphospholipid syndrome, and thrombotic microangiopathic process should be ruled out. Corticosteroids are the main modality of treatment. IVIG can be used in severe cases. Splenectomy was found to be less effective in ITP associated with SLE compared to primary ITP. Control of disease activity with immunosuppressive therapy can be helpful in some cases associated with active disease flares in SLE patients.

## 1. Introduction

Systemic lupus erythematosus (SLE) is an autoimmune disorder characterized by a variety of hematologic manifestations. Thrombocytopenia is one of the diagnostic hematological criteria for SLE. According to ACR (American College of Rheumatology) criteria [1] and SLICC (Systemic Lupus International Collaborating Clinics) criteria [2], thrombocytopenia is defined as platelet count100000/mm^3^ without any obvious cause. It is usually mild in SLE patients and seen in 25 to 50% of SLE patients. Most common cause of thrombocytopenia in SLE is ITP. Severe thrombocytopenia is relatively uncommon in SLE patients and characterized by petechiae, purpura, and bleeding.

## 2. Classification

ITP seen without any apparent associated condition is defined as primary ITP, which accounts for 80% of cases. Secondary ITP is seen in conjunction with another condition. These include infections such as HIV, hepatitis C, H. pylori, CMV, and COVID-19 [3]. Interestingly, cases were identified secondary to vaccination [4, 5]. Similarly, autoimmune diseases such as systemic lupus erythematosus (SLE) and rheumatoid arthritis (RA) and malignancies like chronic lymphocytic leukemia (CLL) and various lymphomas are also responsible for ITP cases.

Patient with secondary ITP were usually significantly older than patients with primary ITP [6]. Furthermore, patients with secondary ITP were usually asymptomatic and did not have significant bleeding manifestations compared with primary ITP. Because of a higher median platelet count and fewer bleeding manifestations, patients with secondary ITP are less likely to require treatment than patients with primary ITP.

## 3. The Development of SLE in ITP Patients

The concern of ITP being primary or secondary was investigated extensively in the previous studies. There is no definitive way to differentiate between primary and secondary ITP in SLE patients. Degree of thrombocytopenia is usually mild to moderate in secondary ITP compared with the primary ITP. Antiplatelet antibodies were reported to be detected in 78% of SLE patients, often without associated thrombocytopenia, and in up to 16% of patients, isolated thrombocytopenia was the initial clinical manifestation [7]. It has been estimated that 3-15% of patients with apparently isolated ITP will develop SLE [8]. Among the 668 patients with ITP, the risk of new-onset ITP was 26.8 times higher [9].

ITP may present before the development of SLE or can present acutely during a disease flare [10]. Severe bleeding from thrombocytopenia is seen in a minority of patients. In a case-control study involving 50 patients with SLE who had thrombocytopenia and 100 controls with SLE and normal platelet counts, thrombocytopenia was associated with greater degrees of organ damage, likely reflecting more active disease [11]. Patients with isolated ITP who are likely to progress to SLE should be identified early because of both prognostic and therapeutic significance [12–14].

Several studies have attempted to identify clinical or laboratory parameters that could possibly predict the development of SLE in ITP patients. Anderson et al. found that 24 of 117 adult patients with ITP (20%) had a positive antinuclear antibody (ANA) titer. Four of them (17%) developed SLE later, and all were women with high titers of ANA [15]. In another study of 82 patients with chronic ITP, 16 patients (20%) had positive ANA and 9 of 16 patients (57%) developed SLE at presentation and shortly thereafter [16]. 12 out of 150 SLE patients (8%) had ITP in a study by Balsalobre Aznar et al. [17]. These studies suggested that high titers of ANA in women with ITP are sensitive but nonspecific marker to predict the development of SLE. However, 29 patients with chronic ITP and ANA positivity did not develop SLE during a 3-year follow-up period [18]. These data suggest that ANA positivity cannot predict the development of SLE in idiopathic ITP cases. Thus, larger studies with further characterization are needed to delineate the possibility of development of SLE in ITP patients.

## 4. Pathogenesis

The mechanisms of ITP in SLE patients have been extensively studied in the previous studies. The predominant mechanism is increased peripheral destruction of platelets mediated by antiplatelet antibodies. Autoantibodies against platelet surface glycoproteins play a pivotal role in destruction of platelet in SLE [19, 20]. Autoantibodies against thrombopoietin or thrombopoietin receptor [21, 22] and antiphospholipid antibodies [23, 24] are found in some cases. Other mechanisms of thrombocytopenia in SLE patients include drug-induced thrombocytopenia, thrombocytopenia associated with splenomegaly, and platelet consumption in the setting of thrombotic microangiopathic process.

Serum platelet-binding IgG and platelet-associated IgG are increased in patients with SLE and thrombocytopenia as in patients with primary ITP [25, 26]. The correlation between the presence of platelet autoantibodies and the different disease manifestations was not statically significant except for thrombocytopenia [27]. But presence of platelet autoantibodies was associated with current thrombocytopenia and disease activity in some patient's [27].

GP2b3a are most frequent in ITP in SLE patients like that of isolated ITP, but they are not specific for thrombocytopenia as these antibodies are found in 30-70% of thrombocytopenic patients, and in contrast, many patients with positive antibodies never develop thrombocytopenia [28, 29]. Antibodies against Gp Ia/IIa, HLA I, and Gp Ib/Ix complex are less frequently isolated [30]. However, some studies showed an almost complete absence of platelet surface glycoproteins in SLE patients [31]. Immunoblot studies showed IgG reactivity to the 50-70 KD internal platelet protein [31].

Multiple studies tried to find out the association between antiphospholipid (aPL) antibodies and thrombocytopenia in SLE patients. One study found that out of 18 patients with SLE and thrombocytopenia, 14 had anticardiolipin (aCL) antibodies and the relative risk for thrombocytopenia in patients with aCL antibodies was greater than four [32]. Another study found that aCL, antiphosphatidic acid, antiphosphoserine, antiphosphatidylinositol, and the lupus anticoagulant were all associated with thrombocytopenia in SLE patients [33]. Membrane phospholipids that are released after cell damage cross react with cardiolipin and are responsible for the anticardiolipin antibodies [34]. It is very important to keep in mind that thrombocytopenia in the setting of antiphospholipid antibodies could be related to consumption of platelets rather than peripheral destruction as seen in SLE patients.

## 5. Diagnosis of ITP in SLE Patients

Patients with SLE usually have some degree of thrombocytopenia. It is very challenging to differentiate whether the thrombocytopenia is secondary to idiopathic ITP vs. secondary ITP as thrombocytopenia can be related to an underlying disease process or the effect of therapies. The pathobiology of secondary ITP is more complex than that of idiopathic ITP. Therefore, a detailed clinical and laboratory evaluation is required before a diagnosis of secondary ITP is made [35].

The diagnosis of secondary ITP is especially difficult in patients taking steroids and immunosuppressive medications for SLE [35]. In patients with thrombocytopenia meeting the diagnostic criteria for SLE, diagnosis of secondary ITP can be made, but other causes of thrombocytopenia such as drug-induced thrombocytopenia, thrombocytopenia associated with splenomegaly, antiphospholipid syndrome, and thrombotic microangiopathic process should be ruled out. Complete blood count, peripheral smear, folate and B12 level, and coagulation parameters including antiphospholipid antibodies are required in the evaluation of thrombocytopenia in SLE patients. Similarly, patients with thrombocytopenia associated with SLE can have anemia and leukopenia as a part of disease spectrum making a diagnosis of secondary ITP more likely.

Thus, a thorough clinical evaluation and laboratory assessment are required to delineate the cause of thrombocytopenia in SLE patients. It is very important to exclude thrombocytopenia as a result of pharmacological agents, which is very challenging.

## 6. Treatment

The management of ITP depends on the severity of thrombocytopenia, presence of symptoms, and other risk factors for bleeding. Severe thrombocytopenia requires urgent treatment with involvement of a rheumatologist. Treatment is usually indicated in individuals who have bleeding manifestations and/or a platelet count less than 30,000/mm^3^. Presence of concurrent risk factors such as anticoagulation treatment, NSAIDs, liver or kidney disease, surgery, and a major trauma should also be considered when considering treatment.

The main principle of treatment is to reduce the risk of bleeding rather than to normalize the platelet count. The risk of bleeding is higher in patients with very low platelet count especially below 10,000, older age, and the presence of other bleeding diathesis. People with these risk factors may require treatment even at higher platelet counts.

Severity of bleeding is generally categorized as follows:

1. Critical bleeding—defined as (a) bleeding in a critical anatomical site including intracranial, intraspinal, intraocular, retroperitoneal, pericardial, or intramuscular with compartment syndrome or (b) ongoing bleeding that results in hemodynamic instability or respiratory compromise [36]
2. Severe bleeding—bleeding that results in fall in hemoglobin of 2 or more g/dL or requires transfusion of 2 or more units of packed RBCs but does not meet criteria for critical bleeding
3. Minor bleeding—bleeding that does not meet criteria for severe or critical bleeding. Skin bleeding or nonsevere mucous membrane bleeding are some examples

Hospitalization is indicated in patients with newly diagnosed ITP and platelet count20000/mm^3^ who are asymptomatic or have minor mucocutaneous bleeding according to the American Society of Hematology 2019 guidelines [37]. Hospitalized patients with critical bleeding require platelet transfusion in addition to treatment with steroids and intravenous immunoglobulin (IVIG). Similarly, patients with severe bleeding require hospitalization and treatment with IVIG or steroids.

Treatment of ITP in SLE is almost similar to that of idiopathic ITP. Some cases associated with SLE disease activity may respond to treatment of underlying SLE. Glucocorticoids or IVIG are the preferred first-line medications for the treatment. Second-line treatments include rituximab, thrombopoietin receptor agonists, splenectomy, danazol, and other immunosuppressive agents.

### 6.1. Corticosteroids

Among corticosteroids, prednisone (0.5 to 2 mg/kg per day) or dexamethasone (40 mg per day for 4 days) is used for the treatment [37]. Prednisone is given as 0.5 to 2 mg/kg per day for one to two weeks with tapering of dose thereafter to complete the total course over less than 6 weeks [37]. Intravenous dexamethasone is preferred for severe or critical bleeding, and dexamethasone can be used orally for minor bleeding or thrombocytopenia without bleeding. Similarly, intravenous methylprednisolone 1 g daily for three days is recommended on severe or critical bleeding. Short course (<6 weeks) of steroids is usually recommended over a prolonged course [37].

Dexamethasone is usually given for four days, and it has advantage of a faster response and no need for drug tapering. On other hand, we can titrate the prednisone dose depending on the patient's response. Dexamethasone was associated with more rapid rise in platelet count at two weeks compared with prednisone (platelet count30000/mm^3^, 79 versus 59 percent) and an almost similar response at six months (54 percent with dexamethasone and 43 percent with prednisone) [38]. If there is no response to the initial dose within 2 weeks, the prednisone should be tapered over 1 week and stopped [39].

Glucocorticoids are associated with serious side effects that are unpredictable. Hyperglycemia, dyslipidemia, hypertension, psychiatric disturbances, and cutaneous effects are some common adverse effects associated with short-term corticosteroid use [40]. Glucocorticoids are the first-line treatment of ITP despite these side effects.

In a study of 59 patients with SLE and thrombocytopenia, 40 responded well to the steroid therapy with an increase platelet count, but only 11 had a sustained response in the mean follow-up period of 78 months [41]. High-dose intravenous methylprednisolone was given to the 10 patients, and 60% had initial response; however, no patient had a sustained response [41]. In another study involving 53 patients, patients were treated with high dose of corticosteroid followed by either cyclophosphamide (17/53) or azathioprine (5/53) with or without intravenous globulin (12/53). Although all patients had a normal platelet count with treatment, 44% patients had at least one relapse during their disease [11]. Thus, corticosteroids are effective in treating thrombocytopenia in most of the patients, but sustained response is unlikely [42].

### 6.2. Intravenous Immunoglobulin (IVIG)

IVIG is used for patients with bleeding, who require rapid increase in a platelet count and in patients unresponsive to corticosteroids [39]. Certain patients with contraindications to steroid (e.g., uncontrolled diabetes, psychiatric disorders, and acute infection) can be managed with IVIG [39]. The platelet count response to the treatment with IVIG is rapid within 12 to 24 hours, and it can be used as a diagnostic criterion for ITP [43]. The effect of IVIG usually lasts for two to six weeks.

The dose of IVIG is 1 g/kg daily for one or two days. Alternatively, 400 mg/kg daily for five days can be used. One randomized trial suggested that initial treatment with 1 g/kg of IVIG was more effective than 0.5 g/kg [44].

In a study of seven patients with thrombocytopenia and SLE who were treated with IVIG, five patients achieved a more than 50% increase in their platelet counts and rise in platelet count was sustained for at least 6 months in four patients [45]. Sixty-three patients with SLE were treated with IVIG, and it was effective mainly for hematologic manifestations, including ITP and improvement of SLEDAI (SLE Disease Activity Index) [46].

IVIG is usually tolerated well in most patients, but it can cause thrombosis [47, 48] and hemolysis [49].

### 6.3. Anti-D

Anti-D can be used in RhD-positive patients as an alternative to the IVIG. Clinicians are not comfortable using anti-D because of risk of hemolytic reaction, and patients should be monitored for signs of hemolysis for four hours after the administration [50]. Anti-D should be avoided in patients with history of hemolysis or a high risk of hemolysis (e.g., positive direct antiglobulin test and elevated reticulocyte count) [51]. Anti-D should be reserved for patients with contraindications or failure of standard therapies. Most of the standard societies do not recommend use of anti-D for treatment of ITP because of low efficacy and side effects.

Anti-D is directed against D antigen of the Rh blood group system, and the main mechanism is the competitive inhibition of the mononuclear phagocytic system by sensitized red blood cells in the spleen [52]. The main mechanism for use in thrombocytopenia is downregulation of FC*γ*RIIIa on splenic macrophages by RBC-specific antibodies [53].

The dose of anti-D is 50 to 75 mcg/kg intravenously, and the dose can be repeated in case of decline in platelet count [54]. Anti-D is usually effective only in patients with an intact spleen [52]. Anti-D has the advantage of a low cost and a longer duration of action over IVIG [55].

### 6.4. Combination Therapy

Combination therapy of steroids and IVIG can be used in patients who fail to respond to a single first-line treatment and very severe cases of bleeding. Some studies found significantly higher response rates with combined therapy compared to the steroid monotherapy [56].

### 6.5. Second-Line Therapies

Second-line therapy is used when first-line therapy does not raise the platelet count to safer levels to prevent bleeding or develop relapse while on the first-line therapy. There are novel therapies that are emerging for the treatment of ITP and shared decision-making considering the cost, ease of administration, potential adverse effects, and efficacy of treatment, all of which may influence patient preference [57].

### 6.6. Splenectomy

Splenectomy is a very effective treatment for ITP as it removes the principal site of phagocytosis of antibody-coated platelets. It was used as the first-line treatment for ITP in the past [58]. Because of the good response rate after the procedure, it is recommended for patients who want to have a single curative procedure. With the widespread availability of other treatment methods with less complication compared to splenectomy, splenectomy is recommended in ITP lasting more than 3 months and in patients who are corticosteroid-dependent or have no response to therapy [37].

Splenectomy has the highest chance of altering the disease course, and most patients achieve sustained remission [59, 60]. In a systemic review including 2623 adults who underwent splenectomy for ITP, a complete response rate of 66% was noted after follow-up for 1 to 153 months [60]. Similar review of 1233 patients showed failure rate of 28% at 5 years for all patients with splenectomy [61]. However, splenectomy has complications related to surgery as well as increased predisposition to infection and vascular events [62].

There are some studies evaluating the effectiveness of splenectomy in SLE patients with ITP. In a study of 25 adults with SLE who underwent splenectomy, 64% patients had sustained complete or partial response rate after a median follow-up of 6.6 years [63]. In another study of 14 patients with SLE who had splenectomies, only 2 patients sustained normal platelet count without need for any additional treatment [64]. Based on the results of these studies, splenectomy may not be a good option for SLE patients with thrombocytopenia.

### 6.7. Rituximab

Rituximab is a good option for patients who prefer not to take medication for prolonged period.

Rituximab is a monoclonal antibody directed against CD20 on the B cell surface which works through different mechanisms including growth regulation, antibody-dependent cytotoxicity, and complement-dependent cytotoxicity [65].

A meta-analysis with five trials including 463 ITP patients showed complete response (platelet count100000/mm^3^) in 47% of patients who were treated with rituximab compared to 33 percent of controls after a median follow-up of 6 months (*p* = 0.02) [66]. In a randomized trial including 112 patients, there was no significant reduction in the long-term treatment failure with rituximab after a median follow-up of 1.5 years, but there was small benefit of prolongation of time to relapse in the rituximab group who attained overall response [67].

Rituximab was used in 261patients with SLE, and thrombocytopenia and reduction of disease activity were noted in almost 50% of patients [68]. A similar study including 10 adult patients with refractory thrombocytopenia treated with a low-dose rituximab for four weeks showed an overall response rate of 50% at 36 weeks [69]. Therefore, rituximab is a second-line agent for refractory thrombocytopenia in SLE patients with a good overall response rate and very few adverse effects.

### 6.8. TPO Receptor Agonists

Thrombopoietin receptor agonists (TPO-RAs) work by stimulating the production of megakaryocytes. They are used as second-line agents for patients not wanting surgery and immunosuppressive effects of medications.

Patient treated with TPO-RAs are usually able to achieve a platelet count to the safe range (>50,000/mm^3^ in most cases), and they may attain transient remissions with safe range of platelet count for several weeks [70]. The platelet count response is usually seen approximately 7 to 14 days after starting these drugs [71]. In one study, 53% of patients had sustained remission after discontinuing eltrombopag without any additional therapy after a median follow-up of 9 months [72]. Platelet count usually drops after the drug is discontinued, and most patients usually require maintenance therapy for long-term benefit.

All TPO-RAs are usually effective for the treatment of ITP. According to ASH guidelines, either eltrombopag or romiplostim can be used, and choice may differ based on patient preference or route of administration [37]. Romiplostim is given as once weekly injection, and eltrombopag is a once daily pill.

Although there is a concern for thrombotic events with use of romiplostim, the thrombosis rate was very minimal, and risk of thrombosis was not associated with platelet count [73]. Similarly, 4% of patients developed thromboembolic events in a study with eltrombopag [74]. A 2015 meta-analysis showed TPO-RAs were associated with a higher risk of thromboembolic events compared with the controls [75].

SLE patients with thrombocytopenia were also treated with TPO-RAs. In a study of three SLE patients with thrombocytopenia treated with eltrombopag, all patients maintained safe range of platelet count (>50,000/mm^3^) for greater than three years following discontinuation of steroids [76]. Another patient with thrombocytopenia and SLE refractory to the conventional therapy achieved a complete response after eltrombopag therapy. Risk of thrombosis should be considered while prescribing TPO-RAs due to combined effect of medication and disease process.

### 6.9. Fostamatinib

Fostamatinib is a spleen tyrosine kinase inhibitor approved for the treatment of chronic ITP in patients without response to previous treatments. Its low stable response rate of 18% makes it unlikely to be used as an alternative to splenectomy, rituximab, or TPO mimetics until direct comparative studies are performed [77].

### 6.10. Immunosuppressive Agents

These medications have low evidence of efficacy and should be reserved for patients who fail first- or second-line treatments.

### 6.11. Azathioprine

Azathioprine is a steroid-sparing agent used in SLE thrombocytopenia and can be used alone or with corticosteroids [78]. In a study involving 53 patients with chronic ITP refractory to other therapies, azathioprine was associated with a good response rate, but few months of treatment are needed to get a response [79].

### 6.12. Mycophenolate Mofetil

Mycophenolate mofetil was used in 46 patients with primary and secondary ITP with a 52% response rate and 33% patients achieving a complete response [80]. There are case reports showing good response with treatment with mycophenolate mofetil in lupus patients with thrombocytopenia refractory to other treatments [81, 82].

### 6.13. Cyclophosphamide

Pulse cyclophosphamide was used in treatment of 20 patients with refractory thrombocytopenia, and 65% achieved a complete response and 20% were able to achieve partial response [83]. Monthly intravenous cyclophosphamide was effective in treating autoimmune thrombocytopenia in patients with SLE refractory to other treatments [84]. Similar studies in SLE patients showed remission of thrombocytopenia with low-dose cyclophosphamide, and low-dose cyclophosphamide followed by azathioprine or mycophenolate mofetil can be effective in inducing remission for severe thrombocytopenia refractory to corticosteroids in SLE [85].

### 6.14. Danazol

Danazol is a synthetic androgen and is used in many types of thrombocytopenia [86, 87]. A systematic review utilizing 38 articles showed effectiveness of danazol in refractory thrombocytopenia in SLE patients, and it is relatively well tolerated [88]. Doses of 600 mg daily have been used, although a low to moderate dose is also being used nowadays and 77.4% patients achieved response within 3 months [89]. It should be taken continuously for 3 months, and prolonged therapy may be needed in some cases [89].

ITP in the setting of SLE is a diagnosis of exclusion after excluding other causes of thrombocytopenia. It is very challenging to diagnose ITP in SLE patients because thrombocytopenia can be associated with multiple factors including the disease process and effect of treatments. It is less severe than primary ITP and is usually treated with same treatment modalities used in primary ITP.

## 7. Conclusions

ITP can be primary with unknown cause or secondary due to an underlying condition. Thrombocytopenia in SLE can be caused by disease process or effect of treatments such as corticosteroids and immunosuppressants. Therefore, diagnosis of ITP can be challenging in SLE patients and requires close coordination between hematologists and rheumatologists to exclude other potential causes of thrombocytopenia. The treatment of ITP in SLE patients is almost similar to the primary ITP, although some studies found that splenectomy may be less effective in these patients.
